# The Rubber Question
*Dr. S. S. White has kindly furnished us with advance sheets of the *Dental Cosmos*, in order that we may give our readers the earliest information of the progress of the suits in the Eastern District of Pennsylvania.—[Ed.]


**Published:** 1873-08

**Authors:** 


### THE RUBBER QUESTION.*

IN ORDER to show the present state of the litigation under the Cummings Patent, we have procured and give below the bill, answer, and replication in one of the several suits now pending in this district.

We have selected a case begun since the decision of the Supreme Court dismissing the Gardner Appeal, in order to show what issues remain open in consequence of that decision.

SAMUEL S. WHITE.

---

UNITED STATES CIRCUIT COURT.
*Eastern District of Pennsylvania.*
IN EQUITY.

*To the Honorable the Justices of the Circuit Court of the United States for the Third Circuit, within and for the Eastern District of Pennsylvania, sitting in Equity.*

THE Goodyear Dental Vulcanite Company, a corporation established by law of the State of New York, and Josiah Bacon, of Boston, Massachusetts, and a citizen of the State of Massachusetts, bring this their bill against William H. Gates, of the City and County of Philadelphia, dentist, and a citizen of the State of Pennsylvania.

*DR. S. S. WHITE has kindly furnished us with advance sheets of the *Dental Cosmos*, in order that we may give our readers the earliest information of the progress of the suits in the Eastern District of Pennsylvania.—[ED.]

And, thereupon, your orators complain and say, that heretofore, to wit, before the seventh day of June, in the year eighteen hundred and sixty-four, one John A. Cummings, who was then a citizen of the United States, being the original and first inventor of a certain new and useful improvement in artificial gums and palates, not at that time in public use, nor on sale with his consent or allowance for more than two years, made application in due form of law to the government of the United States for its letters-patent for such improvement; upon which application such proceedings were had that, on the said seventh day of June, letters-patent of the United States, dated the said seventh day of June, signed by the Secretary of the Interior, and countersigned and sealed with the seal of the Patent Office, by the Commissioner of Patents, were issued and delivered to the said Cummings, for the said invention, entitled an "Improvement in artificial gums and palates," whereby there was granted to the said Cummings, his heirs, administrators, or assigns, for the term of seventeen years from the said date thereof, the full and exclusive right and liberty of making, constructing, using, and vending to others to be used, the said improvement, as in and by a certified copy of the said letters-patent now in court produced, and shown to your honors, will more fully appear.

And your orators further show unto your honors, that afterward, to wit, on the thirteenth day of July, A. D. 1864, by certain deeds of assignment duly executed and recorded in the United States Patent Office, all right, title, and interest in the said letters-patent, and the invention and improvement secured thereby, became vested in the Dental Vulcanite Company, a corporation established by law of Massachusetts, as by the said deeds of assignment, or certified copies thereof, with the endorsements of the recording thereon, now produced and shown to your honors, will more fully appear.

And your orators further show unto your honors, that the said Dental Vulcanite Company, after such assignment to them, surrendered their aforesaid patent, according to law, on account of a defective specification; and a new patent was duly issued to them on the tenth day of January, 1865, for the same invention, in accordance with their corrected description and specification, whereby was granted and secured to the said Dental Vulcanite Company and their successors and assigns, for the full term of seventeen years from the said seventh day of June, 1864, the full and exclusive right and liberty of making, constructing, using, and vending to others to be used, the said improvements therein described and claimed, and hard rubber plates embracing the same, as in and by said reissued letters-patent, or a certified copy thereof, here in court to be produced, will more fully appear.

And your orators further show unto your honors, that afterward, to wit, on the twenty-first day of March, 1865, these last named letters-patent were, according to law, surrendered by the said Dental Vulcanite Company, and canceled on account of defects still discovered in the specification, and a new patent was duly issued, bearing said last-named date, in accordance with their corrected description and specification, and for the same improvements, whereby was granted and secured to the said Dental Vulcanite Company and their successors and assigns, for the full term of seventeen years from the said seventh day of June, 1864, the full and exclusive right and liberty of making, constructing, using and vending to others to be used, the said improvements therein specified and claimed, and hard rubber-plates embracing the same, as in and by said second reissued patent, or a certified copy thereof, here in court to be produced, will more fully appear.

And your orators further show, that on the seventeenth day of July, A. D. 1866, the said Dental Vulcanite Company, by a certain deed of assignment of that date, here in court to be produced, did sell and assign to your orator, Josiah Bacon, of Boston, Massachusetts, the said letters-patent and all the rights thereby vested, together with the right to sue for and collect to his own use all damages for past infringements thereon, and that said assignment was made in trust for certain persons then associated together with intent to form the corporation which is your orator in this bill, and in contemplation of the formation of such corporation, and that such associates did afterward form and are now members of such corporation.

And your orators further show unto your honors, that your orator, the said Bacon, on or about the seventh day of January,

1868, by his deed of that date, here in court to be produced, in pursuance of said trust assigned to your orators, the said Goodyear Dental Vulcanite Company, the said letters-patent, and all rights and privileges under the same, and also the right to demand, sue for, and collect to their own use, all damages at law and profits in equity for past infringements of said letters-patent; but your orators, the said Goodyear Dental Vulcanite Company, submit that they are entitled in equity to recover such profits by reason of the said Bacon's holding of said patent having been in trust as aforesaid, as well as because they are assignees of all said Bacon's right to recover such profits.

And your orators further show unto your honors, that the said Cummings, his mesne assignees, and your orators have always, since the grant of the said letters-patent, and during the time they each owned the same respectively, and in the exercise of the full and exclusive right and liberty so granted as aforesaid, made, used, and vended to others to be used, the said improvement so patented, and have had and maintained, until the infringement hereafter complained of, exclusive possession of the said improvement and invention, under and by virtue of said letters-patent, and have never acquiesced in any invasion or infringement of their said rights.

And your orators further show unto your honors, that since the date of the said letters-patent, more than five thousand dentists, within the limits of the United States, have submitted to the claims of your orators and their assignors under said letters-patent, and have taken licenses to use the said improvement under the same.

And your orators further show unto your honors, that said Dental Vulcanite Company filed their bill of complaint against one Isaac J. Wetherbee on the twenty-fifth day of October, 1864, in the Circuit Court for the District of Massachusetts, praying for an injunction and other relief in equity against said Wetherbee, for infringement of said patent, to which bill of complaint the said Wetherbee filed his answer, denying the validity of said letters-patent, and alleging, among other defenses, that said Cummings was not the original and first inventor thereof, and setting up that many other persons, both in Europe and in the United States, used and practiced said invention before said Cummings's alleged invention. Whereupon such proceedings were had, that evidence was taken by both parties upon all the points at issue, and the case was fully heard and argued before the said Circuit Court, and thereupon, to wit, at the May term, 1866, of said Circuit Court, said court rendered its decision upon all the points at issue, and ordered judgment and a final decree in favor of the complainants, and a perpetual injunction to be issued against said Wetherbee, enjoining him from infringing upon said letters-patent.

And your orators further show unto your honors, that in the latter part of 1866, a large number of dentists combined together to resist said patent in the district of Maryland, and test cases were made on bills filed on November 23, 1866, in the United States Circuit Court for said district, by Josiah Bacon *vs.* Hugh Arthur and Robert Arthur, and by Josiah Bacon *vs.* Adalbert Volck, and a full hearing was had on motions for interlocutory injunctions, on which the validity of said patent was fully and exhaustively argued by counsel for the respective parties, and on the 10th day of December 1866, the court, Giles, J., rendered an opinion in favor of complainants, and decided to grant the injunctions. And thereupon such proceedings were had that decrees were made in favor of complainants in both of said suits, and said injunctions were made perpetual.

And your orators further show unto your honors, that on the 14th day of April, 1868, a bill was filed in the United States Circuit Court for the Western District of Pennsylvania, by the complainants in this cause against Charles Sill and Milton E. Gillespie, for infringement of said letters-patent; and, after full argument by counsel for the respective parties, on a motion for an interlocutory injunction in said suit, the said court, McCandless, J., entered a decision in favor of complainants, and granted the said injunctions, on the 18th day of May, 1868. And thereupon such proceedings were had that on the 13th day of November, 1868, a decree in favor of the complainants was made in said suit, and said injunction was made perpetual.

And your orators further show unto your honors, that one Benoni E. Gardner, a citizen of the State of Rhode Island, infringed upon said patent, whereupon your orators filed their bill of complaint against said Gardner on the 16th day of September, 1868, in the United States Circuit Court for the District of Rhode Island, praying for an injunction and other relief in equity against said Gardner; to which said bill of complaint said Gardner filed his answer, denying the validity of said letters-patent, and alleging, among other defences, that said Cummings was not the original and first inventor of said improvement, and setting up that many other persons had used and practiced the said improvement before the invention thereof by said Cummings, as claimed and set forth in said bill of complaint. And thereupon evidence was fully and exhaustively taken by both parties upon all the points at issue, and the cause was brought to a final hearing on its merits, and fully argued before said court, before Hon. Nathan Clifford, Judge, and thereupon at the June term, 1870, the judgment of said court was pronounced in favor of the complainants on all the points at issue, and a decree was entered, directing among other things that a perpetual injunction be issued against said Gardner, enjoining him from infringing upon said letters-patent. And thereupon the said Benoni E. Gardner appealed from said decree to the Supreme Court of the United States, and the said Supreme Court, after full argument by counsel for both parties, rendered its decision, affirming said decree, on the 6th day of May, 1872.

And your orators further show unto your honors, that the *respondent* herein, having full knowledge of the premises, and in violation of your orators' exclusive right and privilege as aforesaid, and utterly disregarding the same, *has*, since the 17th day of July, 1866, and before and since the said assignment of the said letters-patent to your orators, the Goodyear Dental Vulcanite Company, without the license of your orators, at Philadelphia, Pennsylvania, and at various other places in the United States, manufactured, used, and sold, and still *continues* to manufacture, use, and sell, many artificial gums and palates, embracing the improvement and invention described in said letters-patent, and so secured to your orators—how many, your orators are unable to say, but pray that the *respondent* may discover and set forth in *his* answer to this bill.

And your orators further show unto your honors, that they will be subject to great and irreparable injury, unless they shall obtain from your honors the relief hereby sought.

To the end, therefore, that the said *defendant* may, if *he* can, show why your orators should not have the relief hereby prayed, and may upon ........................................ *oath* ................and according to the best and utmost of *his* ........................................ ........................................knowledge, remembrance, and belief, full, true, direct, and perfect answer make to all and singular the matters hereinbefore stated and charged, as fully and particularly as if the same were hereinafter repeated, and *he* ........................................ thereunto distinctly interrogated, and more especially that *he* ........................................ may answer and set forth,—

1. Whether *he has* not made, or caused to be made, or used, or sold, dental plates made wholly or partly of hard rubber or vulcanite, since the 17th day of July, 1866, and what number thereof, according to the best of *his* knowledge, information, and belief, as near as *he* can approximate the same?

2. Whether *he has* not made, or caused to be made, or used, or sold, dental plates made wholly or partly of hard rubber or vulcanite, since the first day of January last, and what number thereof, according to the best of *his* knowledge, information, and belief, as near as *he* can approximate the same?

3. Whether *he has* not made, or caused to be made, or used, or sold, dental plates made wholly or partly of hard rubber or vulcanite, since the 17th day of July, 1866, in each of the successive years ending on the 31st days of December thereof respectively, specifying separately each of the said years in which any of said dental plates were so made, used, or sold, and what number thereof in each of said years respectively, according to the best of *his* knowledge, information, and belief, as near as *he* can approximate the same?

4. Whether *he has* not received money or property directly or indirectly from the manufacture, use, or sale, since the 17th day of July, 1866, of dental plates made wholly or partly of hard rubber or vulcanite, and what amount of such money or property *he has* received, or *is* entitled to receive from such manufacture, use, or sale, since the 17th day of July, 1866, specifying the amount received in each of the successive years ending on the 31st day of December thereof respectively, and also the amount received since the first day of

January last, according to the best of *his* knowledge, information, and belief, as near as he can approximate the same?

5. Whether *he has* not now on hand a quantity, and how much of any substance composed wholly or partly of gum, or rubber prepared to be manufactured into plates, or gums of hard rubber, or vulcanite?

6. Whether *he has* not now on hand many, and how many dental plates, or gums made wholly or partly of hard rubber or vulcanite?

And that the said *respondent* may answer the premises, and may be decreed to account for and pay over to your orators all such gains and profits as have accrued to *him* from *his* said infringement by making and selling such artificial gums and palates, and also to pay to your orators, in addition to said profits, the damages which have been sustained by your orators by reason of said infringement, and that *he* may be restrained by an injunction issuing out of this honorable court, from making, using, or vending to others to be used, any artificial gums or palates, embracing the improvement so secured to your orators as aforesaid, and also that an injunction issue so restraining the *defendant* pending this cause, and that the said rubber or vulcanite, and the artificial gums and palates so manufactured by *him* of said rubber or vulcanite, and now in possession of said *respondent*, may be destroyed or delivered up to your orators, and for such other and further relief as the nature of the case may require, and to your honors may seem meet.

May it please your honors to grant unto your orators not only a writ of injunction conformable to the prayer of this bill, but also the writ of subpœna directed to said William H. Gates, commanding him to appear and answer to this bill of complaint, and to do and receive what to your honors shall seem meet.

And your orators will ever pray.

GOODYEAR DENTAL VULCANITE COMPANY,
*By its Treasurer, Josiah Bacon.*

J. E. SHAW, *Complainant's Solicitor.* } JOSIAH BACON.

GEO. HARDING, *Of Counsel.* }

UNITED STATES OF AMERICA, } ss.

DISTRICT OF MASSACHUSETTS. }

At Boston, in the county of Suffolk, in said District, this twenty-seventh day of February, A. D. 1873, personally appeared Josiah Bacon, who signs the foregoing bill, and made oath that he has read the same, and understands the same and the contents thereof; and that all the matters therein stated as of the knowledge of the complainants, are to his knowledge true, and as to all other matters therein stated, he verily believes them to be true.

Before me,

{ NOTARIAL SEAL. } E. ERNEST CADUC,

*Notary Public.*

A true copy.

SAMUEL BELL, *Clerk Circuit Court U. S.,*
*Eastern District of Pennsylvania.*

---

UNITED STATES CIRCUIT COURT,
*Eastern District of Pennsylvania.*
IN EQUITY.

THE GOODYEAR DENTAL VULCANITE COMPANY, AND JOSIAH BACON,
*vs.*
WILLIAM H. GATES.
} No. 3, April Session, 1873.

THE answer of William H. Gates, of Philadelphia, Pennsylvania, defendant, to the bill of complaint of the Goodyear Dental Vulcanite Company, and Josiah Bacon, complainants.

This defendant, now and at all times hereafter, saving and reserving to himself all and all manner of benefit or advantage of exception and otherwise, which can or may be had or taken to the manifold errors, uncertainties, imperfections, and insufficiencies in the said bill of complaint contained, for answer thereto, or to so much and such parts thereof as this defendant is advised it is material or necessary for him to make answer unto, answering, saith:

That he is not informed, otherwise than by the allegations in said bill of complaint, and does not know whether the said complainant, The Goodyear Dental Vulcanite Company, is a corporation established by law of the State of New York; and that due proof thereof may be made in this cause, he denies that it is such corporation or so established as alleged in said bill of complaint.

This defendant is informed and believes, and thereupon admits, that before the seventh day of June, in the year one thousand eight hundred and sixty-four, to wit, on or about the twenty-fifth day of March, in said year one thousand eight hundred and sixty-four, one John A. Cummings, who was then a citizen of the United States, made application to the Government of the United States for its letters-patent for a certain alleged new and useful improvement in artificial gums and plates, upon which application such proceedings were had, that on the seventh day of June, in said year one thousand eight hundred and sixty-four, letters-patent of the United States, numbered forty-three thousand and nine (43,009), dated the said seventh day of June, signed by the Secretary of the Interior, and countersigned by the Commissioner of Patents, and sealed with the seal of the Patent Office, were issued and delivered to the said Cummings for the said alleged improvement.

But this defendant, upon information and belief, denies that said Cummings was the original and first inventor of said alleged improvement; he denies that said alleged invention was not at the time of said Cummings's application for said letters-patent in public use and on sale; he denies that it had not been at that time in public use or on sale with his consent or allowance more than two years; and he denies that said Cummings or his assigns have any right to the exclusive use of said alleged improvement.

And this defendant further says upon information and belief, that prior to the said seventh day of June, in the year one thousand eight hundred and sixty-four, the said Cummings had by an instrument of writing, under seal, duly executed and delivered, and dated on or about the first day of March, in said year one thousand eight hundred and sixty-four, assigned, sold, set over, and conveyed unto G. H. P. Flagg and H. D. Osgood, both of Boston, Massachusetts, one undivided quarter of all his right and interest in said alleged invention, and in the said letters-patent issued upon the application therefor, as aforesaid, as, recourse being had to said instrument of writing, as recorded in the United States Patent Office, will more fully and at large appear, and as will fully appear recourse being had to a duly authenticated copy of said instrument, or to said instrument of writing itself, which this defendant is informed and believes is in the possession or under the control of this complainant, Josiah Bacon, and which instrument this defendant therefore prays said Bacon may be required to produce and file in this court, to be used on any examination of witnesses in this cause, and at the hearing thereof, as this defendant may be advised. And this defendant further says upon information and belief, that prior to the said seventh day of June, in the year one thousand eight hundred and sixty-four, the said Cummings had by an instrument of writing, under seal, duly executed and delivered, and dated on or about the sixteenth day of May, in said year one thousand eight hundred and sixty-four, assigned, sold, set over, and conveyed to Joseph Gavett, of Boston, Massachusetts, one undivided eighth part of all his right or property in said alleged invention, and in the said letters-patent issued upon the application therefor, as aforesaid, as will fully appear, recourse being had to said instrument of writing, which this defendant is informed and believes has never been recorded in the United States Patent Office, but which, as he is informed and believes, has been and now is in the possession of or under the control of this complainant, Josiah Bacon, and which instrument of writing this defendant therefore prays said Bacon may be required to produce and file in this court, to be used on any examination of witnesses in this cause, and at the hearing thereof, as this defendant may be advised.

As to the alleged deeds of assignment mentioned in said bill, whereby, on the thirteenth of July, in the year one thousand eight hundred and sixty-four, the said letters-patent and the alleged invention and improvements secured thereby are alleged to have become vested in the Dental Vulcanite Company named in said bill, this defendant is not otherwise informed, and has no knowledge, and he therefore denies the allegations thereof contained in said bill, and leaves the complainants to make proof thereof as they may be advised.

And further answering, this defendant is informed and believes, and thereupon admits, that the aforesaid letters-patent numbered forty-three thousand and nine (43,009) were on or about the twenty-first day of December in said year, one thousand eight hundred and sixty-four, surrendered and canceled, and a new patent numbered (reissue) one thousand eight hundred and forty-eight (1,848) issued on the tenth day of January, in the year one thousand eight hundred and sixty-five, to the Dental Vulcanite Company; but this defendant upon information and belief denies all and singular the other allegations in said bill contained, respecting the surrender of said original letters-patent and the reissue thereof, under date of January tenth, A. D. 1865.

And further answering, this defendant is informed and believes, and thereupon admits, that after said tenth day of January, in the year one thousand eight hundred and sixty-five,—to wit, on or about the twenty-seventh day of February, in the year one thousand eight hundred and sixty-five,—the aforesaid reissued letters-patent numbered one thousand eight hundred and forty-eight were surrendered and canceled, and he so as aforesaid admits that a new patent was thereupon issued to the Dental Vulcanite Company, bearing date the twenty-first day of March, in said year one thousand eight hundred and sixty-five, which new patent is numbered (reissue) one thousand nine hundred and four; but he so as aforesaid denies that said last-mentioned reissue was for the same alleged improvement, and he further denies that thereby was secured to said Dental Vulcanite Company, their successors and assigns, the exclusive right and liberty of making, constructing, using, and vending to others to be used, the alleged improvements therein specified and claimed, and hard rubber plates embracing the same.

And further answering, this defendant says that he is not informed otherwise than by the allegations in said bill of complaint, and does not know whether the said Dental Vulcanite Company did on the seventeenth day of July, A. D. 1866, by a deed of assignment of that date, sell and assign to this complainant, Josiah Bacon, in trust for others, the said letters-patent and all the rights thereby vested, together with the right to sue for and collect to his own use all damages for past infringements thereon; and therefore, and in order that due proof may be made in that behalf as the complainants may be advised, this defendant denies each and every of the allegations in said bill contained respecting such alleged assignment.

And further answering, this defendant says that he is not informed otherwise than by the allegations in said bill of complaint, and does not know whether the said Bacon, on or about the seventh day of January, 1868, by his deed of that date, assigned to this complainant, the Goodyear Dental Vulcanite Company, the said letters-patent and all rights and privileges under the same, and also the right to sue for and collect to their own use all damages at law and profits in equity for past infringement of said letters-patent; and therefore, and in order that due proof may be made in that behalf as the complainants may be advised, this defendant denies each and every of the allegations in said bill contained respecting such alleged assignment.

And this defendant denies, upon information and advice, that said complainant, the Goodyear Dental Vulcanite Company, are entitled in equity to recover profits, as alleged in the said bill of complaint, by reason of said Bacon's alleged holding of said patent having been in trust, as alleged in said bill, as well as because they are alleged assignees of all said Bacon's alleged right to recover such profits.

And further answering, this defendant, upon information and belief, denies that the said Cummings, his mesne assignees, and these complainants, have always, since the grant of said letters-patent and during the time they each owned the same respectively, and in the exercise of the alleged right and liberty so granted as aforesaid, made, used, and vended to others to be used, the said alleged improvement so patented as aforesaid; or have had and maintained, until the infringement complained of in said bill, exclusive possession of said alleged improvement and invention under and by virtue of said letters-patent; and he so as aforesaid further denies that they have never acquiesced in any invasion or infringement of their said alleged rights.

And further answering upon information and belief, this defendant further denies that since the date of the said letters-patent, more than five thousand dentists within the limits of the United States have submitted to the claims of these complainants and their assignors under said letters-patent, and have taken licenses to use the said alleged improvement.

And this defendant, further answering, says that he is informed and believes, and thereupon admits, that on or about the twenty-fifth day of October, A. D. 1864, the said Dental Vulcanite Company filed their bill of complaint against one Isaac J. Wetherbee in the United States Circuit Court for the District of Massachusetts, praying for an injunction and other relief in equity against said Wetherbee for alleged infringement of said original letters-patent numbered forty-three thousand and nine, and dated June 7, A. D. 1864, as aforesaid; that the said Wetherbee filed his answer to said bill of complaint, denying the validity of said letters-patent, and alleging, among other defenses, that said Cummings was not the original and first inventor of the improvement patented to him in said original letters-patent No. 43,009, and setting up that many other persons, both in Europe and in the United States, used and practiced said invention before said Cummings's alleged invention.

And this defendant, further answering upon information and belief, says that upon the filing of said answer to said bill by said Wetherbee, or very soon thereafter, the said letters-patent were surrendered and canceled and reissued under date of January 10, 1865, and this reissue again was thereafter surrendered and canceled and reissued under date of March 21, A. D. 1865, as hereinbefore set forth. That after said several reissues of said letters-patent, said cause was proceeded in between said Dental Vulcanite Company and said Wetherbee, but the pleadings therein on the part of said Wetherbee were in the final decision of said suit adjudged irregular; and under such adjudication many of the defenses set up by said Wetherbee were excluded from consideration by said court; others of the defenses set up by said Wetherbee were avoided by the reissue of said letters-patent after said Wetherbee had filed his answer to said bill of complaint; others of said Wetherbee's defenses in said suit were not supplemented by proof, but were waived or abandoned, and the only points at issue on the final hearing of said cause did not involve the defenses hereinafter made by this defendant, nor include the proofs upon which this defendant relies; and the decree alleged in the bill of complaint as rendered in said suit was not based upon correct conclusions of law or fact, and is therefore not in anywise conclusive as to the validity of the said reissued letters-patent set out in the bill of complaint in this cause, nor as to the rights of the complainants alleged therein.

And further answering, this defendant says that respecting the alleged proceedings in the United States Circuit Court for the District of Maryland by Josiah Bacon against Hugh Arthur and Robert Arthur and against Adalbert Volck, and respecting the alleged proceedings in the United States Circuit Court for the Western District of Pennsylvania against Sill and Gillespie, mentioned in said bill of complaint, this defendant has no knowledge or information other than is afforded by the said bill of complaint itself, save that he is informed and believes that all of said proceedings were taken and had under or in connection with the letters-patent granted to Nelson Goodyear, May 6, 1851, as reissued in 1858, and said suits were not, nor was any or either of them, brought to final hearing, nor any proofs taken therein after the interlocutory injunctions were granted, as alleged in said bill of complaint; and he is further informed and believes that said suits were not contested suits, and did not raise any inquiry into the validity of the letters-patent set out in the bill of complaint in this cause, and that said suits were compromised, or the defenses thereof abandoned, in view of their connection with the aforesaid letters-patent of Nelson Goodyear; and this defendant is advised and believes, and thereupon avers, that said proceedings are not in anywise conclusive as to the validity of the said reissued letters-patent set out in the bill of complaint in this cause, nor as to the rights of the complainants alleged therein.

And further answering, this defendant says that respecting the proceedings in the United States Circuit Court for the District of Rhode Island, by these complainants, The Goodyear Dental Vulcanite Company and Josiah Bacon, against Benoni E. Gardner, as alleged in said bill of complaint, and respecting the appeal taken from the decree of said Circuit Court to the Supreme Court of the United States, and the affirmance of said decree by said Supreme Court on the 6th day of May, 1872, he is informed and believes and thereupon avers that said proceedings in said suit in the Circuit Court of Rhode Island, and in the said Supreme Court of the United States, were collusive, fraudulent, and false; that there was no dispute between said complainants and said Gardner, in said Circuit Court nor in said Supreme Court; that the decree in said Circuit Court was not and was not intended to be enforced, but that before and at the date of such decree said Gardner was protected against the same by agreements made in that behalf between these complainants and one J. B. Newbrough; that the injunction awarded against said Gardner was not and was not intended to be enforced; that the costs in said suit and on said appeal were paid by these complainants; that both sides of said cause were argued, at the fraudulent and collusive hearing thereof before Mr. Justice Clifford, by counsel who were paid for such arguments by these complainants; that these complainants paid counsel on both sides for preparing the record in said cause and for arguing the same on the appeal before said Supreme Court, and that both in said Circuit Court and in said Supreme Court these complainants controlled the preparation and argument of the said cause so as to make the same collusive in both courts; that upon due proceedings had in that behalf these facts were laid before the said Supreme Court of the United States, at the adjourned term in October, 1872, and that in their answer, filed in the course of these proceedings, these complainants admitted that in said suit they had paid the counsel employed for the pretended defense, as well as for themselves, in the Circuit Court, and subsequently in said Supreme Court, and thereupon the said Supreme Court of the United States did, as this defendant is informed and believes, on or about the third day of March, A. D. 1873, annul and vacate its affirmance of said decree in Rhode Island, and dismissed the said false and collusive appeal, and recalled the mandate which had been issued to said Circuit Court, by reason of the said cause having been merely collusive both in the court below and in the said Supreme Court, so that said decrees are not in anywise conclusive as to the validity of the said reissued letters-patent set out in the bill of complaint in this cause, nor as to the rights of the complainants alleged therein.

And for answer to the interrogatories in said bill contained, this defendant says, to the first of said interrogatories, that he has made, or cause to be made, and has sold dental plates made wholly or partly of hard rubber or vulcanite since the seventeenth day of July in the year one thousand eight hundred and sixty-six.

To the second of said interrogatories, this defendant, answering, says that he has made, or caused to be made, and has sold dental plates made wholly or partly of hard rubber or vulcanite since the first day of January last. To so much of said first and second interrogatories as demands the number of such plates made, or caused to be made, and sold by this defendant, and to the whole of the third and fourth of said interrogatories, this defendant respectfully suggests, by way of objection, exception, and demurrer, that these complainants are not or are not at this stage of this cause entitled to answers to said parts of said first and second interrogatories, nor to any part of said third and fourth interrogatories, for the following, among other reasons, to wit: that said parts of said first and second interrogatories, and the whole of said third and fourth interrogatories, are irrelevant and impertinent, and not founded upon any sufficient averment in said bill of complaint; that without an ascertainment and adjudication that this defendant has violated or infringed the reissued letters-patent mentioned in said bill, and until it is decreed that the said reissued letters-patent are good and valid, the complainants are not entitled to the aforesaid information demanded in said interrogatories; and that in advance of a decree for an account, the complainants are not entitled to inquire into the defendant's business, or to demand the particulars or incidents thereof.

To the fifth of said interrogatories this defendant answers No.

To the sixth of said interrogatories this defendant says that he does not keep on hand any dental plates or gums made wholly or partly of hard rubber or vulcanite, but has them made to order only, and delivers them as soon as completed, and has none now on hand.

And this defendant, still insisting upon each and all of the foregoing objections to the bill of complaint, and to the aforesaid reissued letters-patent numbered one thousand nine hundred and four, further answering says, by way of further defenses to said bill of complaint, that he is informed and believes, and upon information and belief avers—

I. That the pretended invention or improvement, or a substantial and material part thereof, or substantial and material parts thereof, described and set forth and claimed in the reissued letters-patent numbered one thousand nine hundred and four, and dated March twenty-first, in the year one thousand eight hundred and sixty-five, was or were not described nor specified nor represented nor suggested nor indicated in the specifications, drawings or model deposited in the United States Patent Office in connection with the application of said Cummings for the original letters-patent numbered forty-three thousand and nine, and dated June 7, A. D. 1864, before the issue of said original letters-patent; nor was nor were such pretended invention or improvement nor such part or parts thereof as aforesaid described or specified or represented or indicated or suggested in the specifications or drawings of said original letters-patent, numbered forty-three thousand and nine, and issued June 7, 1864, but that the said pretended invention and improvement, or the said substantial and material part or parts thereof, was or were first described and set forth and claimed by the said Cummings in the new or amended and corrected description and specification annexed to the reissued letters-patent (granted and issued upon the surrender and cancellation of said original letters-patent) numbered one thousand eight hundred and forty-eight, and dated January tenth, in the year one thousand eight hundred and sixty-five, and that said pretended invention and improvement, or said substantial and material part or parts thereof, was or were again described and set forth and claimed by the said Cummings in the new or amended description and specification annexed to the reissued letters-patent (granted and issued upon the surrender and cancellation of said first-mentioned reissued letters-patent No. 1,848, dated January 10, 1865), numbered one thousand nine hundred and four, and dated March 21, 1865, by reason of which facts said letters-patent numbered (reissue) one thousand nine hundred and four, and dated March 21, 1865, were granted and issued contrary to law, and were and are null and void.

II. That the claim set forth by said Cummings in the new or amended and corrected description and specification annexed to the reissued letters-patent No. 1,904, dated March 21, 1865, and granted as mentioned in said bill of complaint, does not cover or comprise any device or invention or improvement which is by law the proper subject of letters-patent of the United States, but that said claim is wholly invalid, void, and inoperative in law.

III. That the said John A. Cummings was not the original and first inventor or discoverer of the thing patented, or of a substantial and material part or of substantial and material parts thereof, claimed as new in said reissued letters-patent numbered one thousand nine hundred and four, and dated March 21, 1865, but that long prior to the pretended invention thereof by said Cummings, the same thing, or a substantial and material part thereof, or substantial and material parts of the thing patented or claimed as new in said reissued letters-patent, was known to and used by the following-named persons, and at the following-named places, to wit:

George E. Hawes, of and at New York City.

C. O. Crosby, of and at New Haven, Connecticut.

William A. Royce, of and at Newburgh, New York.

Elias Wildman, of and at Philadelphia, Pennsylvania.

Charles Goodyear, deceased, formerly of New York City, at said New York City, and at New Haven, Connecticut, and in London, England, and Paris, France.

Charles Goodyear, Jr., of New Rochelle, New York, at New York City, and at New Haven, Connecticut, and at London, England, and Paris, France.

Asa Hill, of and at Norwalk, Connecticut:

Emanuel G. Blackman, of and at Norwalk, Connecticut.

C. S. Putnam, deceased, at New York City.

L. E. Christopher, of and at New York City.

William A. Bevin, of and at Boston, Massachusetts.

F. A. Bevin, deceased, at New Haven, Connecticut.

G. A. Foster, of and at Utica, New York.

Henry B. Goodyear, of and at New Haven, Connecticut, and at New York City.

Willard Ball, of and at Walpole, New Hampshire.

Nelson Goodyear, deceased, of and at New York City, and at New Haven, Connecticut.

Jehial Parmley, of and at New York City.

John Lovejoy, of New York City, and if not deceased, of New York City.

Archibald McIlroy, of Greenwich, Connecticut, and at New York City.

IV. That the said John A. Cummings was not the original and first inventor or discoverer of the thing patented, or of a substantial and material part or of substantial and material parts thereof, claimed as new in said reissued letters-patent No. 1,904, dated March 21, 1865, but that anterior to the supposed discovery thereof by said Cummings the thing patented, or a substantial and material part thereof, or substantial and material parts of the thing patented or claimed as new in said reissued letters-patent No. 1,904, had been described in the following printed publications, to wit:

*The Lancet.* A Journal of British and Foreign Medical and Chemical Science, Criticism, Literature and News. MDCCCXLV. In Two Volumes Annually. Volume II. Edited by Thomas Wakley, Surgeon, M.P. for the Metropolitan District of Finsbury, and Coroner for the County of Middlesex. London: Printed for the Editor and Published by John Churchill, Princes Street, Soho. In the number of said work for September 13, 1845, pp. 284, 285, 286, 287 of said volume, and in the number of said work for September 20, 1845, pp. 310, 311, 312 of said volume.

*The American Journal of Dental Science.* Edited by Chapin A. Harris, M.D., D.D.S., etc. New Series, Vol. I. Philadelphia, Lindsay & Blakiston; Baltimore, Armstrong & Berry; New Orleans, John Ball. 1850. Pages 3 to 12 (both inclusive) of said volume, being the number of said journal for October, 1850. Said *American Journal of Dental Science*, Volume IV., New Series, at pages 79 to 93; also at page 176; and also at pages 223–226, being respectively the numbers of said journal for October, 1853, and for January, 1854. Also in said *American Journal of Dental Science*, Volume VI., New Series, at pages 477, 478 and 479, being the number of said journal for July, 1856.

*The Dental News Letter.* Vol. IV., January, 1851, No. 2. Jones, White & McCurdy, Proprietors and Publishers, 120 Arch Street, Philadelphia; 263 Broadway, New York, and Branch at 23 Tremont Row, Boston; pp. 59, 60, 61, 62 and 63 of said number of said publication.

*The Dental News Letter.* Edited by J. D. White and J. R. McCurdy. Published Quarterly by Jones, White & McCurdy, Proprietors and Publishers, 528 Arch Street, Philadelphia; 335 Broadway, New York, and Branch at 16 Tremont Row, Boston. Volume XI., April, 1858, No. 3; pp. 170, 171 and 172 of said number of said publication. Also in said *Dental News Letter* for July, 1858 (being No. 3 of said Vol. XI.,) at pages 253, 254 and 255, and on the second page of the advertisements at the back of said number of said work. Also in said *Dental News Letter* for July, 1859, (being No. 4 of Vol. XII.,) at pages 289 and 290 of said number of said publication.

*The Vulcanite.* A Quarterly Journal, devoted to the Science of Mechanical Dentistry. Edited by B. W. Franklin, New York. Published by the American Hard Rubber Co., No. 640 Broadway. Vol. I., No. 1, May, 1860, pages 2 to 14 (both inclusive;) Vol. I., No. 2, August, 1860, pages 65, 66, 67, 68, 69, and 70; also Vol. I., No. 3, November, 1860, at pages 99, 100, 101, 102, 103, 104 and 105; also pages 130 to 132; also Vol. I., No. 4, February, 1861, pages 164 to 168, and pages 178, 179 and 180; also Vol. II., No. 1, May, 1861, pages 32 to 36, pages 40 to 43; also Vol. II., No. 2, August, 1861, pages 106 to 110; also Vol. II., No. 3, November, 1861, pages 142 to 146; also Vol. II, No. 4, February, 1862, pages 176 to 181.

*The Principles and Practice of Dental Surgery.* By Chapin A. Harris, M.D., D.D.S., etc., etc. Philadelphia: Lindsay & Blackiston. Fifth Edition, 1853. Pages 736, 737, 738, 739 and 740.

*A Dictionary of Medical Terminology, Dental Surgery and the Collateral Sciences.* By Chapin A. Harris, M.D., D.D.S., etc., etc. Second edition. Philadelphia: Lindsay & Blakiston, 1855; at pages 343 and 344, under the title "Gutta-Percha."

*Great Exhibition of the Works of Industry of all Nations,* 1851. *Official Descriptive and Illustrated Catalogue.* In three volumes. Vol. I., London, 1851; at p. 474. Article No. 720.

*Exhibition of the Works of Industry of all Nations,* 1851. *Reports of the Juries on the Subjects in the Thirty Classes into which the Exhibition was divided.* London, 1852; at p. 598.

*The American Journal of Dental Science.* New Series. Volume II., at page 332; being the number of said journal for January, 1852.

*Comptes Rendus Hebdomadaires des Seances de l'Academie des Sciences.* Publies conformement a une Decision de l'Academie en date du 13 Juillet, 1835. Par MM. les Secretaires Perpetuels. Tome trente-troisieme, Juillet-Decembre, 1851. Paris: Bachelier, Imprimeur-Libraire de l'Ecole Polytechnique au Bureau des Longitudes, etc., Quai des Augustins, No. 55; 1851; at page 126.

*The Dental Register of the West.* Published Quarterly, by order of the Mississippi Valley Association of Dental Surgeons. Cincinnati; John D. Thorpe, Printer, No. 12 West Fourth St., 1850; at pp. 38, 39 and 40, being the number of said Register for October, 1850.

*The New York Dental Recorder.* Devoted to the Theory and Practice of Surgical, Medical and Mechanical Dentistry. Edited by C. C. Allen, M.D., Dentist. Volume IV. New York: Printed by Grossman & Son, 59 Ann St. 1850; pages 286, 287 and 288, being the number of said journal for September, 1850.

Also, that the pretended invention or improvement described and set forth and claimed in said reissued letters-patent No. 1,904, or a substantial and material part thereof, or substantial and material parts thereof claimed as new, had been long prior to the alleged invention or discovery of said Cummings described in the following letters-patent and application for letters-patent of the United States, to wit: Letters-patent No. 8,075, granted to Nelson Goodyear, of New York city, dated May 6, 1851. Reissued letters-patent No. 556 and reissued letters-patent No. 557, granted to Henry B. Goodyear, administrator of Nelson Goodyear, deceased, each dated May 18, A.D. 1858; being reissues of said letters-patent No. 8,075. Letters-patent No. 9,968, granted to Charles Goodyear, of New Haven,

Connecticut, assignee of Charles Goodyear and Robert Haering, dated April 12, 1853. The rejected application of Robert Haering, of New York, New York, for letters-patent for a new and improved mode of inserting artificial teeth; filed in the United States Patent Office, March 25, 1856. Letters-patent No. 8,621, granted to John Allen, of Cincinnati, Ohio, dated December 23, 1851. Letters-patent No. 12,156, granted to Sharpless Clayton, of West Chester, Pennsylvania, dated January 2, 1855; letters-patent No. 16,708, granted to Alfred A. Blandy, of Baltimore, Maryland, dated March 3, 1857; letters-patent No. 19,916, granted to George Diffenbach, of New York, New York, dated April 13, 1858; letters-patent No. 10,847, granted to Mahlon Loomis, of Cambridgeport, Massachusetts, dated May 2, 1854; and also patented or described in the following English letters-patent, to wit:

**No. 11,135, dated March 18, 1846, to Thomas Hancock.**
**No. 11,208, dated May 15, 1846, to Charles Hancock.**
**No. 11,209, dated May 15, 1846, to H. V. Bartlett.**
**No. 12,241, dated August 15, 1851, to E. T. Truman.**
**No. 13,542, dated March 4, 1851, to A. V. Newton.**
**No. 24, dated October 1, 1852, to Moses Poole.**
**No. 43, dated October 1, 1852, to Moses Poole.**
**No. 577, dated March 14, 1855, to Charles Goodyear, Jr.**
**No. 893, dated April 21, 1855, to Henri Schoofs.**
**No. 1,232, dated May 1, 1857, to Alfred A. Blandy.**
**No. 193, dated January 21, 1859, to James Childs.**
**No. 1,842, dated August 9, 1859, to F. L. Lawrence.**
**No. 2,295, dated October 8, 1859, to James Childs.**
**No. 2,790, dated December 8, 1859, to John Macintosh.**
**No. 4, dated January 2, 1860, to H. A. Dewar.**
**No. 1,549, dated June 25, 1860, to Mathew Cartwright.**
**No. 1,421, dated June 11, 1859, to G. C. Ash.**

V. That the pretended invention or improvement described and set forth and claimed in said last-mentioned reissued letters-patent numbered one thousand nine hundred and four, and dated March 21, 1865, as hereinbefore set forth, or a substantial and material part thereof, was, or substantial and material parts thereof were, in public and common use among dentists and on sale to dentists and others and by dentists and others throughout the United States, at the time of and before the application for said reissued letters-patent last mentioned, and had so been in public and common use and on sale for more than two years prior to such application, and that the said pretended invention or improvement described and set forth and claimed in said last-mentioned reissued letters-patent, or a substantial and material part thereof was, or substantial and material parts thereof were, in public and common use among dentists and others, and on sale to dentists and others and by dentists and others throughout the United States, at the time of and before the application for the original letters–patent numbered forty-three thousand and nine, and dated June 7, 1864, as hereinbefore set forth; and had so been in public and common use and on sale for more than two years prior to such application for said original letters-patent; and such use and sale as aforesaid was had, *inter alia*, by the following–named persons and at the following-named places respectively, to wit:

Clarke S. Putnam, deceased, of and at New York City.

Bradley W. Franklin, of and at the City of New York.

The American Hard Rubber Company of Bethany, Connecticut, and Flushing, New York, and New York City.

Henry B. Goodyear, of and at New Haven, Connecticut.

E. E. Crofoot, of and at Hartford, Connecticut.

A. W. Todd, of and at Montgomery, Alabama.

James Taylor, of and at Cincinnati, Ohio.

Hawes, Bro. & Seabury, of and at Providence, Rhode Island.

T. H. Burras, of and at New York City.

Spencer Roberts, of and at Philadelphia, Pennsylvania.

A. E. Pursell, of and at Madison, Indiana.

S. S. Blodgett, of and at Ogdensburg, New York.

C. Palmer, of and at Warren, Ohio.

R. Russell, of and at Nashville, Tennessee.

Miller & Hale, of and at Rockford, Illinois.

John Mc[illegible]alla, of and at Lancaster, Pennsylvania.

F. A. Bevin, deceased, at Walpole, New Hampshire, and at Boston and Cambridge, Massachusetts, and at New Haven, Connecticut.

Ira A. Salmon, of and at Boston, Massachusetts.

Willard Ball, of and at Walpole, New Hampshire.

Nathan C. Keep, of and at Boston, Massachusetts.

Roswell Cutler, of and at Boston, Massachusetts.

Samuel Mallett, of and at New Haven, Connecticut.

Elbridge G. Leach, of Stoughton, Massachusetts, at said Stoughton, and at Boston, and at Cambridgeport, Massachusetts.

Seth P. Miller, of and at Worcester, Massachusetts.

Dennis S. Bartlett, of and at Boston, Massachusetts.

Ambrose Lawrence, of and at Lowell, Massachusetts.

VI. That (still denying that said Cummings was the original or first inventor of the pretended invention or improvement described and set forth and claimed in said reissued letters-patent numbered one thousand nine hundred and four) the pretended invention or improvement described and set forth and claimed in said last-mentioned reissued letters-patent, or a substantial and material part thereof, or substantial and material parts thereof, had been in public use and on sale with the consent and allowance of said Cummings for more than two years before his application for said reissued letters-patent, and for more than two years prior to his application for said original letters-patent numbered forty-three thousand and nine, as hereinbefore set forth; and such use and sale thereof was had and made as aforesaid, with the consent and allowance and with the knowledge and acquiescence of said Cummings, among others, by the following-named persons, and at the following-named places, to wit:

F. A. Bevin, deceased, late of Boston, Massachusetts, at said Boston, at Cambridge, Massachusetts, and at New Haven, Connecticut, and elsewhere.

W. A. Bevin, of New York, at Boston, Massachusetts.

N. C. Keep, of and at Boston, Massachusetts.

I. A. Salmon, of and at Boston, Massachusetts.

Seth P. Miller, of and at Worcester, Massachusetts.

Roswell Cutler, of and at Boston, Massachusetts.

Ambrose Lawrence, of and at Lowell, Massachusetts.

B. W. Franklin, of and at New York City.

The American Hard Rubber Company, of Flushing, New York, and of Bethany, Connecticut, and of New York City, at New York City and elsewhere.

VII. That (still denying that said Cummings was the original and first inventor of the pretended invention or improvement described and set forth and claimed in said reissued letters-patent numbered one thousand nine hundred and four) the said Cummings, previous to the date of said reissued letters-patent, and previous to the date of his application therefor, and previous to the date of the reissued letters-patent numbered one thousand eight hundred and forty-eight, as hereinbefore set forth, and previous to his application therefor, and previous to his application for said original letters-patent No. 43,009, suffered the said pretended invention and improvement or the substantial and material part or parts thereof described and set forth and claimed in the amended and corrected specification signed by him, and annexed to said reissued letters-patent numbered one thousand eight hundred and forty-eight, and again described and set forth and claimed in said reissued letters-patent numbered one thousand nine hundred and four, to be used freely and fully by the public for more than two years, and acquiesced in and permitted and assented to such use without asserting any claim or right thereto, and thereby waived and abandoned the same, and dedicated the same to public use, and surrendered and forfeited or lost any right he might have had in or to said pretended invention or improvement, or to letters-patent of the United States therefor, or under such letters-patent.

VIII. That by English letters-patent, numbered 577, and dated March 14, A. D. 1855, granted and issued to Charles Goodyear, Jr., then residing in Paris, France, the pretended invention or improvement described and set forth, and claimed in said reissued letters-patent numbered one thousand nine hundred and four, and dated March 21, 1855, or a substantial and material part, or substantial and material parts of said pretended invention or improvement, was or were patented in a foreign country more than six months prior to the application for said reissued letters-patent last mentioned, and more than six months prior to the application, as hereinbefore set forth, for the reissued letters-patent numbered one thousand eight hundred and forty-eight, and more than six months prior to the application, as hereinbefore set forth, for the original letters-patent numbered forty-three thousand and nine, and that the same had been introduced into public and common use in the United States (as hereinbefore more particularly set forth,) prior to the application for said reissued letters-patent, respectively, and prior to the application for said original letters-patent, and more than two years prior to said applications, respectively, for said reissued letters-patent numbered one thousand eight hundred and forty-eight and one thousand nine hundred and four, and more than two years prior to said application for said original letters-patent numbered forty-three thousand and nine, wherefore said reissued letters-patent numbered one thousand nine hundred and four were and are null and void.

IX. That (still insisting upon the aforesaid defenses and each of them) the pretended invention or improvement described and set forth and claimed in said reissued letters-patent numbered one thousand nine hundred and four, and dated March 21, 1865, or a substantial and material part, or substantial and material parts, of said pretended invention or improvement having been so as last aforesaid patented in a foreign country, more than six months prior to the application for said original letters-patent numbered forty-three thousand and nine, said letters-patent numbered forty-three thousand and nine, and dated June 7, 1864, and said reissued letters-patent numbered one thousand nine hundred and four, and dated March 21, 1865, were by the Acts of Congress in such cases made and provided limited to the term of seventeen years from the date or publication of said foreign letters-patent granted to Charles Goodyear, Jr., as last mentioned, and therefore ceased to be of any force or effect on or before the fifteenth day of March in the year one thousand eight hundred and seventy-two, or on or before the ninth day of September in said year one thousand eight hundred and seventy-two, according as the date or publication of said foreign letters-patent shall be held to have been the fourteenth day of March or the eighth day of September in said year one thousand eight hundred and fifty-five.

X. That as matter of fact, the aforesaid foreign letters-patent No. 577, so granted as aforesaid to said Charles Goodyear, Jr., were dated March 14, A.D. 1855, and expired under the provisions of the laws of England in such cases made and provided, and under the conditions contained in said letters-patent, on or before the fourteenth day of March in the year one thousand eight hundred and fifty-eight, and that, as matter of law, the said fourteenth day of March, A.D. 1855, is to be deemed and taken in this cause as the date or publication of said foreign letters-patent, and that the term of said original letters-patent numbered forty-three thousand and nine, and of said reissued letters-patent numbered one thousand nine hundred and four, could only have lawfully been for seventeen years from said fourteenth day of March, A.D. 1855, and that said term of said reissued letters-patent therefore expired on or before the fourteenth day of March in the year one thousand eight hundred and seventy-two, and before the filing of the bill of complaint in this cause, and that said reissued letters-patent numbered one thousand nine hundred and four had, for the foregoing reasons, ceased to be of any force or effect whatever before the filing of the said bill of complaint.

XI. That the schedule or specification and claim of said Cummings annexed to and forming part of said reissued letters-patent numbered one thousand nine hundred and four, is informal, insufficient, and void in law, and does not and cannot entitle the said Cummings or his assigns or legal representatives to any exclusive use, possession, or enjoyment of the pretended invention or improvement described and specified in said schedule or specification and claim; and the said Cummings or his assigns or legal representatives cannot hold or enjoy the exclusive right or title to said pretended invention or improvement by virtue of his said reissued letters-patent. That the pretended invention or improvement described and set forth and claimed in the said reissued letters-patent, numbered one thousand nine hundred and four, consists only in the substitution of a material well known and in public use long prior to the pretended invention or discovery of said Cummings for other materials which had long theretofore been known and used for the same purposes, in the same mode, and by the same means described in said reissued letters-patent, or in filling the long theretofore well-known moulds with the long theretofore well-known preparations of india rubber or caoutchouc instead of with metal or other materials, and subjecting the long theretofore well-known material to the long theretofore well-known process of vulcanization in the long theretofore well-known way.

And this defendant, still insisting that such substitution of hard rubber or vulcanite, or its equivalent, was not first invented by said Cummings, but had long prior to his pretended invention been known and used by the persons and at the places specified in the foregoing clauses of this answer marked III. and V., and described in the printed publications specified in the foregoing clause of this answer marked IV., further insists that even if said Cummings were the original and first inventor of such substitution of a plate of vulcanite for the plates of different materials theretofore used, such substitution of one material for another is not patentable subject matter for letters-patent of the United States, and that therefore said reissued letters-patent numbered one thousand nine hundred and four were and are null and void.

XII. That if the pretended invention or improvement described and set forth and claimed in the said reissued letters-patent numbered one thousand nine hundred and four embraces or comprises the means or mode or method of obtaining the impression of the mouth and of preparing the moulds and of setting the teeth in the moulds, or the method of making and using said hard rubber or vulcanite plates set forth in said reissued letters-patent, then said reissued letters-patent were and are null and void, for the following among other reasons:

First. Because said Cummings was not the original and first inventor of such means or mode or method of obtaining the impression of the mouth, or of preparing the moulds, or of setting the teeth in the moulds, or of the method of making and using said hard rubber or vulcanite plates, set forth in said reissued letters-patent, but the same or substantially the same means or modes or methods had been known and used and patented, and described in public works long prior to such supposed invention by said Cummings, by the persons and at the places and in the printed publications specified in the foregoing clauses of this answer, marked III., IV. and V.

Second. Because such pretended invention or improvement, so described, set forth, and claimed in said reissued letters-patent, was not described, suggested, or indicated in the specification or drawings of said original letters-patent numbered forty-three thousand and nine, but was new matter interpolated in said reissued letters-patent numbered one thousand nine hundred and four, such interpolation of said new matter in said reissued letters-patent making a radical change in the specification and in the pretended invention or improvement described and set forth and claimed in said reissued letters-patent from the pretended invention or improvement described and set forth and claimed in said original letters-patent numbered forty-three thousand and nine.

Without this that any other matter or thing in the said complainant's bill of complaint contained material or necessary to make answer unto, and not herein and hereby well and sufficiently answered, traversed, confessed and avoided, or denied, is true to the knowledge or belief of this defendant.

All which this defendant is willing to aver, maintain, and prove as this honorable court shall direct, and humbly prays to be hence dismissed with his reasonable costs and charges in this behalf most wrongfully sustained.

W. H. GATES.

---

UNITED STATES OF AMERICA,<br>EASTERN DISTRICT OF PENNSYLVANIA, } *ss.*

William H. Gates, the above-named defendant, being duly sworn, says that he has read the foregoing answer by him subscribed, and knows the contents thereof, and that the same is true of his own knowledge except as to matters which are therein stated to be on his information or belief, and as to those matters he believes them to be true.

W. H. GATES.

Subscribed and sworn to before me,<br>this 12th day of May, A.D. 1873. }

{ NOTARIAL SEAL. } FRANK M. ETTING,<br>*Notary Public.*

IN THE CIRCUIT COURT OF THE UNITED STATES,
*In and for the Eastern District of Pennsylvania,*
IN THE THIRD CIRCUIT.

GOODYEAR DENTAL VULCANITE COMPANY et al. *vs.* WILLIAM H. GATES. — C. C. U. S. In Equity.

The replication of the Goodyear Dental Vulcanite Company and Josiah Bacon, complainants, to the answer of William H. Gates, defendant.

These repliants, having and reserving unto themselves all and all manner of advantage or exception to the manifold errors and insufficiencies of the said answer for replication thereunto, say that they will aver, maintain and prove their said bill to be true, certain and sufficient, in the law to be answered unto, and that the said answer of the said defendant is uncertain, untrue, and insufficient to be replied unto by these repliants: without this that any other matter or thing in said answer contained material or effectual in the law to be replied unto, confessed and avoided, traversed or denied, is true, all of which matters and things these repliants are and will be ready to prove as this honorable court shall direct, and humbly pray as in and by their said bill they have already prayed.

For complainants,

GEO. HARDING,
J. E. SHAW,
*Solicitors.*

True copy.

[L. S.] SAMUEL BELL, *Clerk Circuit Court U. S., Eastern District of Pennsylvania.*

